# Epidemiology of childhood enterovirus infections in Hangzhou, China

**DOI:** 10.1186/s12985-015-0294-4

**Published:** 2015-04-14

**Authors:** Wei Li, Xiao Zhang, Xi Chen, Yu-Ping Cheng, Yi-Dong Wu, Qiang Shu, Xue-Jun Chen, Shi-Qiang Shang

**Affiliations:** Department of Clinical Laboratory, Children’s Hospital of Zhejiang University School of Medicine, 3333 Binsheng road, Hangzhou, 310003 China

**Keywords:** Enterovirus, Epidemiology, Children

## Abstract

**Background:**

There are over 100 serotypes of enterovirus species A-D, which are the common cause of various symptoms in infants, such as meningitis, encephalitis and hand foot mouth disease (HFMD). This study aims to investigate the epidemiological characteristics of enteroviruses in Hangzhou, Zhejiang province, China, and to provide relevant information to guide public health responses and interventions.

**Methods:**

Systematic surveillance was conducted on enterovirus infections. Samples were collected from children admitted to the inpatient wards and outpatient departments between January 2010 and December 2012 in the Children’s Hospital, Zhejiang University School of Medicine. Enteroviruses from all specimens were detected by RT-PCR using a commercialized detection kit.

**Results:**

From 13026 samples collected and examined, 2673 (21.21%) were found positive for enteroviruses. The annual enterovirus-positive rate decreased from 32.78% in 2010 to 14.23% in 2012. Positivity rate for enteroviruses was highest among children aged less than 5 years. The monthly positivity rate for enterovirus infection ranged from 2.6% to 34.83%, with a peak in June and July. Serotypes causing severe symptoms such as HFMD including EV71 and CA16 were decreasing, while the proportion of unidentified EV serotypes causing herpangina and viral encephalitis were on the rise.

**Conclusions:**

EV infection is highly prevalent among young children in Hangzhou, as it is in the most other parts of the world. Further surveillance using methods that can subtype all EVs is warranted to better monitor these infections and their etiology.

## Background

Enteroviruses (EVs) are single-stranded RNA viruses which belong to the Picornaviridae family. There are over 100 serotypes and can be divided into human enterovirus A to D, non-human enteroviruses and rhinoviruses [1]. Human enteroviruses cause various clinical manifestations, such as neurological, cutaneous, respiratory and visceral diseases [2,3]. In particular, there was a large scale outbreak of hand foot mouth disease (HFMD) in 2008 in Anhui province, China. Since then, large outbreaks of HFMD occurred annually in China [1-6]. Many studies have shown the epidemiological features and pathological mechanisms of enterovirus 71 (EV71), coxsackievirus A16 (CA16), coxsackievirus A6 (CA6) and coxsackievirus A10 (CA10) which are the major subtypes of EVs contributing to HFMD [3-9]. Few studies have simultaneously investigated the epidemiological characteristics of all EVs in children in Hangzhou, Zhejiang province, China, while epidemiology of enterovirus infections in children was well defined in other countries [10-13]. In order to better define and supplement the epidemiology of EV infections in Hangzhou, East China, we conducted systematic surveillance of EV infections in 13026 children from Hangzhou, China.

## Results

### Patients’ characteristics

From January 1, 2010 to December 31, 2012, a total of 13026 samples were collected and tested for enteroviruses, including 11348 from outpatients and 1678 from hospitalized children. Among them, 7795 samples were from boys and 5231 were from girls, yielding a male-to-female ratio of 1.49:1. 2673 were tested positive for enterviruses, with a positivity rate of 21.21%. Among the 2673 EV-positive samples, 1587 were from boys and 1086 were from girls, giving positivity rates of 20.36% in boys and 20.76% in girls.

### The epidemiological characteristics of enterovirus infection

To compare the annual positivity rates of EV infections, we forced surveillance year by year with 3112 tests in 2010, 4467 in 2011 and 5447 in 2012. The positivity rates markedly decreased (*P* < 0.05) by year with 37.28% in 2010, 19.66% in 2011, and 14.23% in 2012.

**Figure 1:**
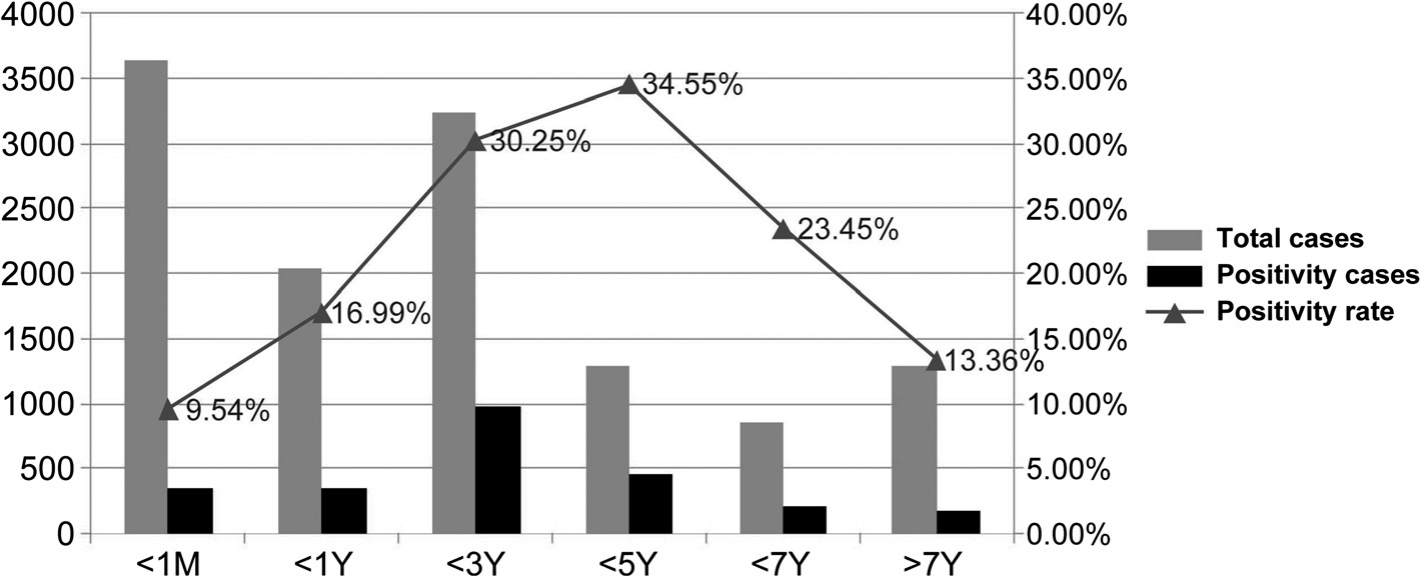
Age distribution of entervirus infections among children younger than 14 years.

Positivity rate for EV infection was at the peak in children aged 1–5 years (30.25%–34.55%, *P* < 0.05), and steadily decline with increase or decrease of age (Figure 1). However, for the children older than 5 years, the positivity rate of EV infection was still as high as 23.45% in 5–7 years and 13.36% in above 7 years. Among all age groups, children younger than 1 month had the lowest infection rate (9.54%, *P* < 0.05). For the children with EV infection, 84.99% were <5 years old, with constitution ratios of 13.92%, 13.84%,39.33%, 17.90%, 8.07% and 6.94% in children aged 0–1 month, 1 month–1 year, 1–3 years, 3–5 years,5-7 years and >7 years, respectively.

**Figure 2:**
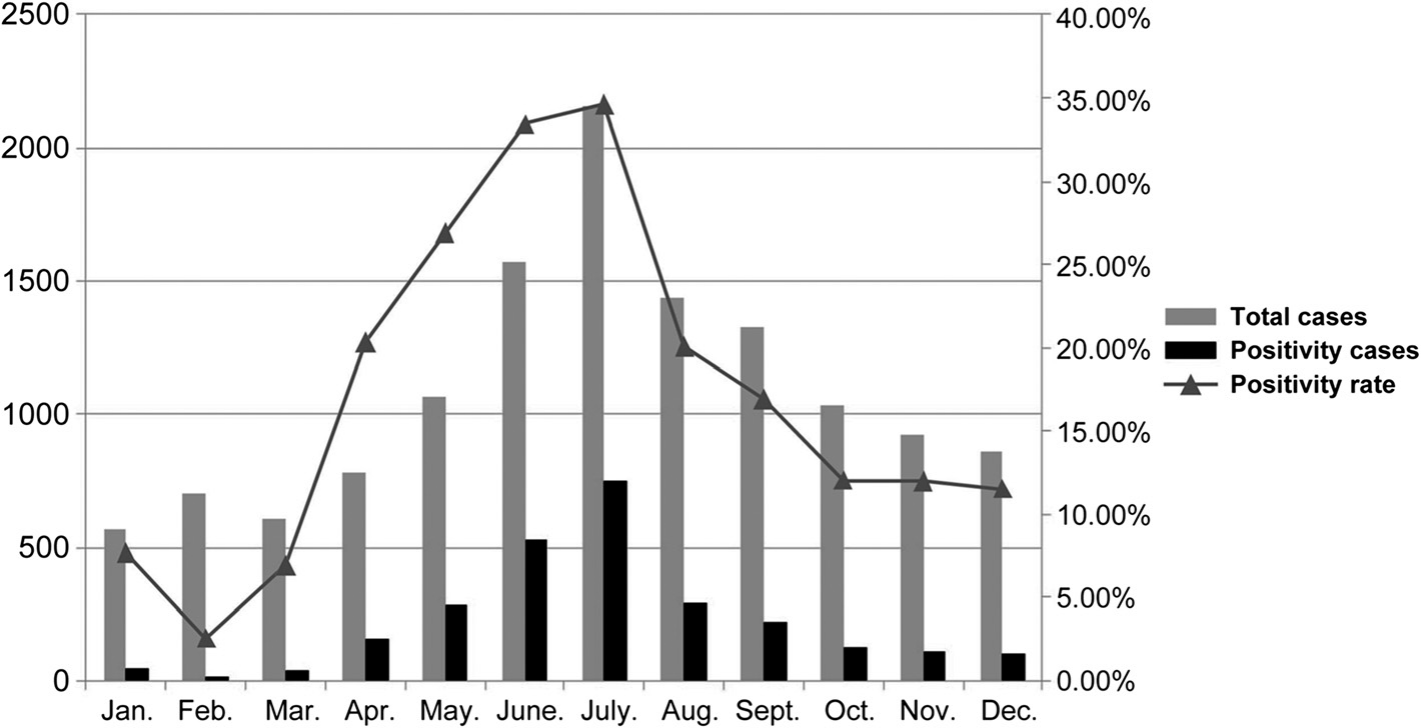
Monthly distribution of entervirus infection among children younger than 14 years.

EV infections occurred year-round, but there was a well-defined seasonality during the 3-year surveillance period. The monthly positive detection rates for EV infections all year round ranged from 2.57% to 34.62%, with a peak in June and July, and steadily declined in the previous and the following months (Figure 2).

**Figure 3:**
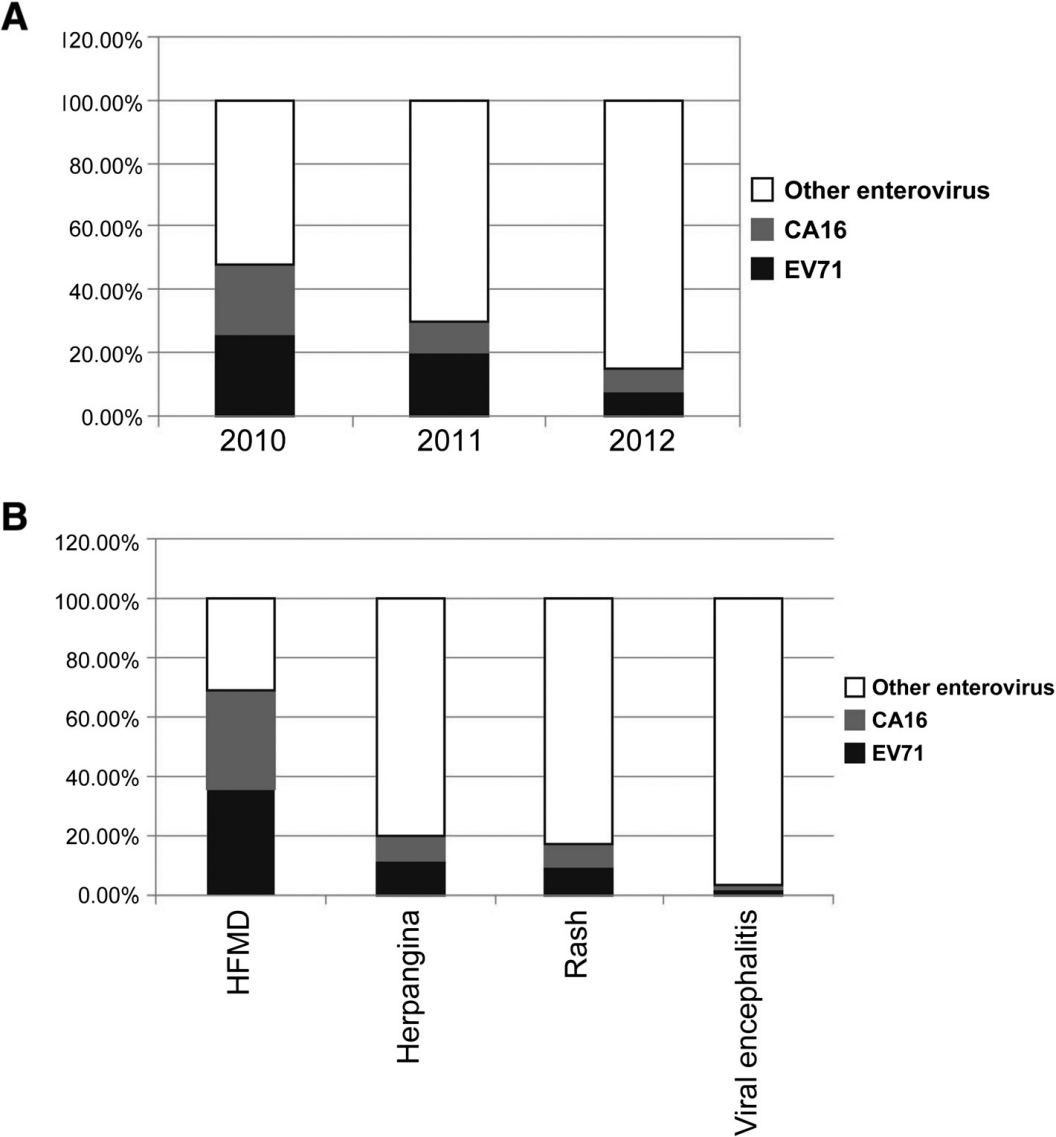
Distribution of EV71, CoxA16 and other enteroviruses among children younger than 14 years. **(A)**. Yearly distribution of EV71, CoxA16 and other enteroviruses; **(B)** Distribution of EV71, CoxA16 and other enteroviruses in enterovirus-associated diseases.

We also estimated distribution of EV71, CoxA16 and other subtype of EVs in EVs positive cases. With increase of age, EV71 and CoxA16 infections decreased among children younger than 14 years (Figure 3A). From 2010 to 2012, the majority of the enteroviruses detected from the HFMD cases were CA16 and EV71 (69.32%), while unidentified enteroviruses were major pathogens contributing to other diseases, with 80.70% of herpangina, 83.08% of rash and 96.96% of viral encephalitis (Figure 3B).

## Discussion

Enteroviruses are the most common viruses infecting human, causing a wide spectrum of illnesses, such as HFMD, herpangina and viral meningitis [14-16]. This is a retrospective study monitoring enteroviruses in children with HFMD, herpangina, viral encephalitis or rash in Hangzhou. During the three-year study period, EV positivity rates in Hangzhou decreased from 32.78% in 2010 to 14.23% in 2012. It may be due to the extremely high incidence of HFMD in 2010, and then lower incidence in the next two years in China [3,6,17]. The proportion of HFMD among EV positive patients are 50.78%, 43.39% and 14.32% in 2010, 2011 and 2012 respectively (not shown in figures). The decreasing of EV positivity rates maybe also due to circulation of EV subtypes changing in Hangzhou with EV71, CA16 decreasing and CA10, CA6 increasing [18].

Our data have shown that summer (May to July) is the peak season for EV infections with the highest incidence in July. The epidemiology data is similar to previous research in HFMD [1]. 1–5 years old children were the most susceptible population with a peak incidence under 5 year of age. Therefore, more attention should be paid to children aged less than 5 years in the summer season for prevention and control of EVs.

The majority of EVs among the HFMD cases were CA16 and EV71 which were predominant in 2008 and 2010 [1-6]. With HFMD prevalenting in 2010, the proportion of EV71 and CA16 is up to 50% in all enteroviruses. Our results also supported that EV71 and CVA16 circulate widely and actively in China as two main causative pathogen strains of HFMD, with the proportion up to 70% among all EV serotypes. The other 30% of EVs causing HFMD may belong to CVA6 and CVA10 as suggested by previous reports [18-23]. Together with the decrease of HFMD cases, the proportions of EV71 and CA16 in all enteroviruses also dropped year by year. On the other hand, with the increase of proportions of other EV strains, the proportions of herpangina, viral encephalitis and rash disease are predicted to increase in the coming years. Our previous study suggested that echovirus 30, 31 and Coxsackie B1 are the most common enteroviruses in these diseases in Hangzhou [16]. Most importantly, in our institute in the year of 2012, 1854 cerebrospinal fluid (CSF) samples were also determined and 109 samples were EV positive, among which 1 case was EV71, none was CA16, but 108 cases were with un-determined EV serotypes(data not shown). Zhang et al. also suggested that echovirus and Coxsackievirus type B are predominant enteroviruses in CSF samples in Zhejiang province [24]. These indications suggest that other subtypes of enterovirus contribute more to herpangina, viral encephalitis and rash disease which require caution of surveillance in the future in Hangzhou. Furthermore, since the profile of prevalent EV strains is changing, lack of clinically surveillance to other serotypes of EVs may hinder the monitoring and controlling of other severe EV infections in Hangzhou. Based on our findings, we suggest the need for systematic surveillance to readily identify more serotypes in the near future in Hangzhou and other part of China.

In this study, commercial real-time RT–PCR assay kit was used to detect enterovirus. The primers and target gene were unidentified which limits our research of sequencing and identifying subtype of enterovirus. In further study, we will amplify and sequence VP1 gene of enterovirus from EV-positive patients which would be helpful to further surveillance of enterovirus.

## Materials and methods

### Patients

Hangzhou is the capital city of Zhejiang Province with the population of 8.84 million. The Children’s Hospital of Zhejiang University School of Medicine is the largest comprehensive center for pediatric health care in Zhejiang province and holds the leading position among Chinese children’s hospitals. The annual outpatient visits sum up to more than 1,000,000 and the annual inpatient visits is nearly 30,000. This hospital also is HFMD treatment and enteroviruses detection listed hospital in Hangzhou in 2010–2012. From January 2010 to December 2012, altogether 13026 children from both the inpatient wards and outpatient departments of our hospital were enrolled in this study, the criteria for inclusion were [1] All of patients who visit Children’s Hospital of Zhejiang University School of Medicine in 2010–2012, [2] age <14 years, primary diagnosis of HFMD, herpangina, viral encephalitis, rash with suspected EV infections. This study was approved by the medical ethics committee of the Children’s Hospital of Zhejiang University School of Medicine (NO.2014-013), and informed consent was obtained from parents.

### Detection of enterovirus

Throat swab from every child was collected from symptomatic children with suspected enterovirus infection. Personal information on demographic factors and medical history were obtained from their guardians by a standard questionnaire. RNAs were extracted from each specimen using Takara MiniBEST Viral RNA/DNA Extraction Kit Ver.5.0 (TAKARA, Japan). The detection of EVs (EV A-D) and further classification of EV71 and CVA16 for EV-positive samples were performed in ABI7500 system by commercial one-step real-time RT–PCR assay kit (Da An Gene Co. Ltd, China) which was recommended by China CDC and was described in previous reports [9,14]. The real time RT-PCR was conducted under this conditions: 15 min at 50°C, 5 min at 95°C, and then followed by 40 cycles of 15 sec at 94°C and 45 sec at 55°C. Samples with CT value less than 35.0 were identified positive.

### Statistical analysis

Statistical analyze was performed using the *X*^2^ test, the statistical significance were calculated using SPSS 17.0 software (SPSS Inc., Chicago, IL, USA).
